# Qihuang needle therapy for Parkinson's disease: a triple-arm randomized controlled trial protocol assessing efficacy and neuroplasticity via multimodal MRI

**DOI:** 10.3389/fneur.2026.1722126

**Published:** 2026-04-13

**Authors:** Wanqing Peng, Yanning Liu, Ziting Huang, Renhui Zhao, Yujie Liu, Zhijuan Liu, Jing Wang, Xinyu Li, Xubo Hong, Xingdong Lin, Jiaming Hong, Nanbu Wang, Zhenhu Chen

**Affiliations:** 1 The First Clinical Medical College, Guangzhou University of Traditional Chinese Medicine, Guangzhou, Guangdong, China; 2 Acupuncture and Rehabilitation Centre, The First Affiliated Hospital of Guangzhou University of Chinese Medicine, Guangdong, China; 3 The Third Affiliated Hospital of Guangzhou University of Chinese Medicine, Guangzhou, China; 4 School of Medical Information Engineering, Guangzhou University of Chinese Medicine, Guangzhou, Guangdong, China; 5 State Key Laboratory of Traditional Chinese Medicine Syndrome, The First Affiliated Hospital of Guangzhou University of Chinese Medicine, Guangzhou, Guangdong, China

**Keywords:** acupuncture, multimodal MRI, neuroplasticity, Parkinson's disease, randomized controlled trial

## Abstract

**Background:**

Parkinson's disease (PD) is a progressive neurodegenerative disorder for which no curative therapy currently exists. Qihuang needle therapy (QNT) is a novel acupuncture technique that has demonstrated clinical promise in alleviating PD symptoms, but its neuroplasticity mechanisms remain unexplored. This trial aims to investigate the efficacy and neurological effects of the QNT via multimodal MRI.

**Methods:**

This triple-arm randomized controlled trial will enroll 69 PD patients randomized into three groups (1:1:1): verum acupuncture, sham acupuncture, or wait-list control. Patients in the verum and sham groups will receive eight treatment sessions over 4 weeks, followed by an 8-week follow-up period. The control group will not receive any acupuncture treatment throughout the trial. Clinical outcomes included the Unified Parkinson's Disease Rating Scale Part III (UPDRS-III), Non-Motor Symptoms Scale (NMSS), 39-item Parkinson's Disease Questionnaire (PDQ-39), muscle rigidity (shear-wave elastography), and gait/balance parameters (Footscan system). Multimodal MRI will evaluate neuroplasticity markers: gray matter volume, functional connectivity, white matter integrity, nigral iron deposition, and neuromelanin content.

**Discussion:**

This protocol pioneers the integration of the QNT with advanced neuroimaging to elucidate its neuroplasticity mechanisms in PD, providing high-level mechanistic evidence for the integration of the QNT into PD care and optimizing integrative treatment strategies that combine traditional Chinese and Western medicine.

**Clinical trial registration:**

(http://itmctr.ccebtcm.org.cn/), Identifier :ITMCTR2025000402.

## 1 Introduction

Parkinson's disease (PD) is the second most prevalent neurodegenerative disorder worldwide, affecting nearly 12 million individuals globally, with its burden projected to rise steadily due to population aging (1). Clinically, PD manifests as resting tremor, rigidity, bradykinesia, and postural instability, underpinned by progressive nigrostriatal dopaminergic degeneration and α-synuclein pathology (2). Although levodopa remains the mainstay of pharmacotherapy, long-term use is limited by many complications and failure to halt disease progression (3). These limitations underscore the need for non-pharmacological approaches, including physical therapy, occupational therapy, and acupuncture, which are recommended by international guidelines to optimize comprehensive PD care (4, 5).

Acupuncture has gained recognition in international guidelines as a complementary therapy for PD (6). Among various techniques, Qihuang needle therapy (QNT) developed from classical *Huangdi Neijing* principles, is distinguished by its unique needle design and operational methodology. A randomized controlled trial showed that QNT significantly improves UPDRS-III scores, gait parameters, muscle rigidity, and non-motor symptoms (7). However, the neurobiological basis of these benefits, particularly the effects on brain network reorganization and neuroplasticity, remains unknown.

Neuroplasticity plays a dual role in PD: adaptive plasticity may initially mitigate symptoms, whereas maladaptive changes may accelerate degeneration (8, 9). Multimodal magnetic resonance imaging (MRI) enables non-invasive assessment of structural and functional brain alterations, providing insights into PD-related neuroplasticity (10). Acupuncture has been shown to modulate brain networks in other neurological conditions, such as improving white matter integrity after mild traumatic brain injury (11), reorganizing motor networks in stroke (12), and modulating key regions in mild cognitive impairment (13). However, despite these promising insights, systematic neuroimaging investigations specifically targeting Parkinson's disease remain scarce. However, no study has systematically applied multimodal MRI to investigate the neuromodulatory and neuroplastic effects of QNT in PD.

This study aims to: (1) conduct a triple-arm randomized controlled trial to evaluate the clinical efficacy of QNT in PD, and (2) integrate multimodal MRI to map functional connectivity reorganization, gray matter volume changes, white matter integrity, nigral iron deposition, and neuromelanin preservation, thereby identifying neuroplasticity targets induced by acupuncture.

## 2 Methods

### 2.1 Study design

This is a parallel, triple-arm, randomized, sham-controlled trial designed to evaluate the clinical efficacy of the QNT and elucidate its neuroplasticity mechanisms in PD. Eligible PD patients will be randomized into three groups (1:1:1): verum acupuncture, sham acupuncture, or wait-list control. The total observation period spans 9 weeks, including a 1-week baseline, a 4-week intervention, and a 4-week follow-up. Additionally, 20 age- and sex-matched healthy controls (HCs) will be recruited to provide normative reference data for baseline MRI comparisons. The HCs data will be used to establish PD-related brain alterations by comparing HCs vs. PD patients at baseline, thereby identifying disease-specific structural and functional abnormalities that may be modulated by QNT.

**Figure 1:**
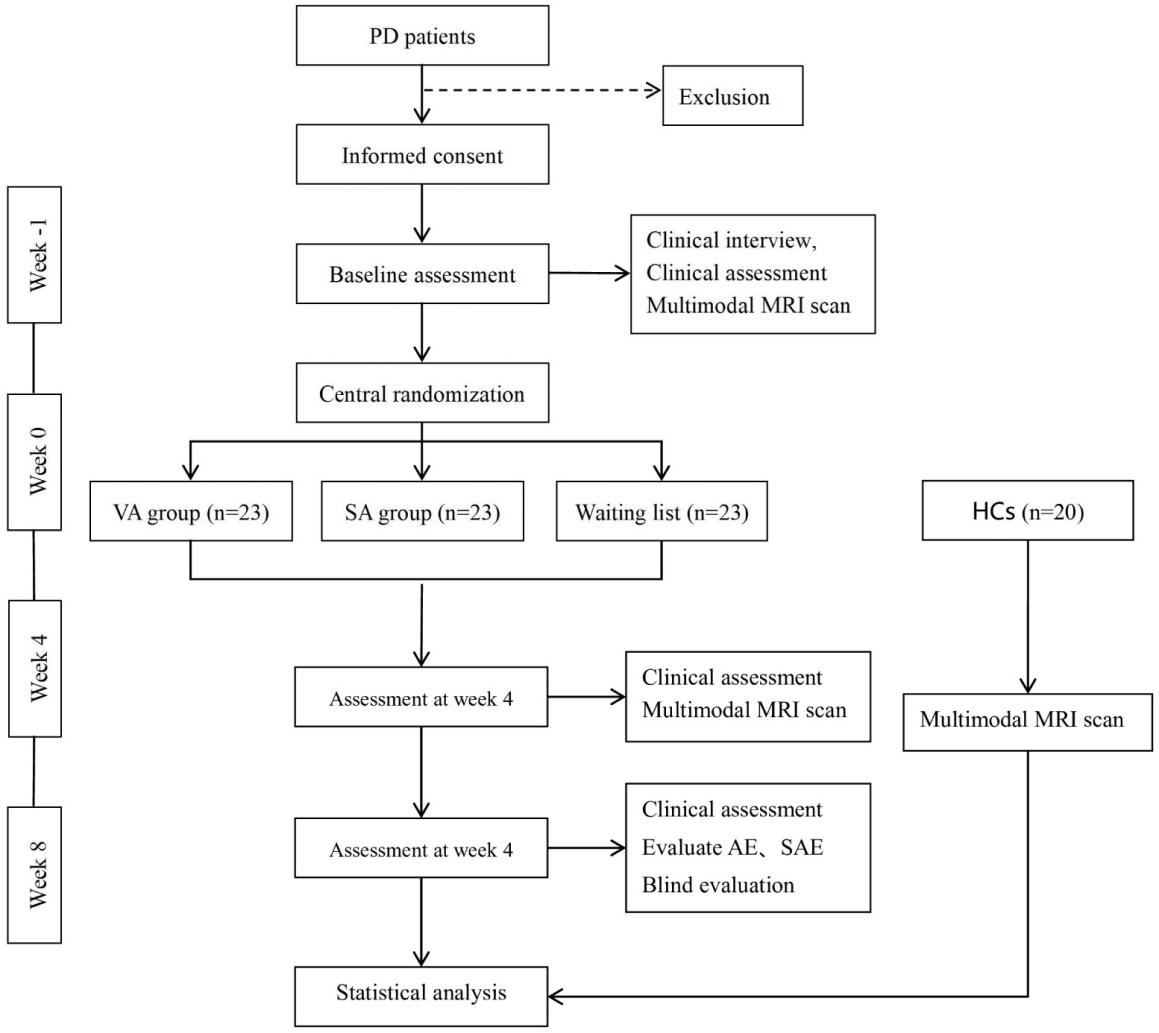
Trial flow chart. PD, Parkinson's disease; VA, Verum acupuncture; SA, sham acupuncture; HCs, healthy controls; MRI, magnetic resonance imaging; AE, adverse events; SAE, serious adverse events.

The protocol adheres to the Standard Protocol Items: recommendations for Intervention Trials (SPIRIT (14) (Supplementary material 1) and the Standards for Reporting Interventions in Clinical Trials of Acupuncture (STRICTA) (15). A flowchart of the study design is shown in Figure 1, and assessment time points are detailed in Table 1.

**Table 1:** Time points of treatment assessment.

Time point	Enrollment	Baseline	Treatment		Follow-up
	Week 1	Week 0	Week 2	Week 4	Week 8
Screening and enrollment					
Clinical interview	×				
Laboratory test	×			×	
Eligibility screen	×				
Informed consent	×				
Randomization		×			
Interventions					
Acupuncture			Eight sessions treatment + basic treatment		
Sham acupuncture			Eight sessions treatment + basic treatment		
Waiting-list			Basic treatment		
Assessments					
UPDRS-III		×		×	×
MDS-NMSS		×		×	×
PDQ-39		×		×	×
Footscan		×		×	
Synapsys		×		×	
SWE		×		×	
Multimodal MRI		×		×	
Others					
AEs, SAEs			×	×	×
Blinding assessment			×^*^	×	
PESA		×			
Patient compliance				×	×
Deqi sensation assessment^**^			×	×	
Medication adherence		×	×	×	×

^*^Assessed after the first and fourth session of acupuncture treatment.

^**^Assessed immediately after each acupuncture treatment.

UPDRS-III, Parkinson's Disease Rating Scale part III motor examination; NNMS, Parkinson's Disease Non-motor Symptom Scale; PDQ-39, 39-item Parkinson's Disease Questionnaire; SWE, shear wave elastic imaging; MRI, magnetic resonance imaging; AE, adverse events; SAE, serious adverse events; PESA, patients expectation scale of acupuncture.

### 2.2 Participants

#### 2.2.1 Inclusion criteria for PD patients

(1) Diagnosis of idiopathic PD according to established diagnostic criteria (16) and no change in the type or dose of anti-parkinsonian medication for at least 4 weeks.
(2) Age 25–80 years;
(3) Hoehn-Yahr stages 1–4.
(4) Patients with no contraindications to MRI or who were willing to undergo imaging.
(5) Conscious, stable vital signs without severe cognitive or auditory impairments.
(6) Signed informed consent.

#### 2.2.2 Exclusion criteria for PD patients

(1) Secondary parkinsonism (e.g., vascular, drug-induced).
(2) MRI contraindications (e.g., metal implants) or severe systemic diseases.
(3) Participation in other clinical trials within the past 3 months.
(4) Severe psychiatric disorders or communication barriers.

#### 2.2.3 Inclusion criteria for HCs

(1) Age 25–80 years; no gender restrictions.
(2) No history of PD, parkinsonian syndromes, or cardiovascular diseases.
(3) No MRI contraindications.
(4) Conscious, stable vital signs, without cognitive or auditory impairments.
(5) Signed informed consent.

#### 2.2.4 Exclusion criteria for HCs

(1) MRI contraindications (e.g., claustrophobia).
(2) Presence of PD-related motor symptoms (e.g., tremor, rigidity).
(3) Cardiovascular diseases (e.g., hypertension, stroke) or severe comorbidities (e.g., cancer, renal failure).
(4) Psychiatric or communication disorders.

### 2.3 Sample size

This trial is designed as an exploratory mechanistic study, with the primary objective of detecting QNT-induced neuroplasticity using multimodal MRI. The sample size was therefore determined based on neuroimaging methodology standards. Given the lack of standardized methods for sample size estimation in acupuncture-related neuroimaging trials, we referenced fMRI studies that recommended ≥20 participants per group for robust statistical analysis (17). Accounting for a 10% attrition rate, 69 PD patients (23 per group) and 20 HCs will be enrolled. HCs undergo baseline MRI only, minimizing dropout risk. Separately, a formal power calculation for the UPDRS-III was performed using PASS 15.0. Based on our pilot data (7), the expected changes (mean ± SD) are: verum acupuncture group 14.0 ± 9.94, sham group 2.84 ± 3.08, and wait-list group 2.3 ± 3.0 (18). With α = 0.05, power = 0.90, 1:1:1 allocation, and Tukey–Kramer correction for multiple comparisons, the required sample size is 165 patients (55 per group) after adjusting for 20% dropout. This trial, however, is designed as an exploratory mechanistic study; the smaller sample of 69 patients is adequately powered for the primary neuroimaging endpoints, whereas all clinical efficacy analyses are considered exploratory and will inform the design of future large-scale multicenter trials.

#### 2.3.1 Recruitment strategy

The study will be conducted from January 2025 to January 2026 at the First Affiliated Hospital of Guangzhou University of Chinese Medicine. Participants will be recruited via hospital advertisements and online platforms.

#### 2.3.2 Written informed consent

Eligible participants will receive detailed written information about randomization (verum/sham/wait-list), potential benefits/risks, and voluntary withdrawal rights. Consent forms (Supplementary material 2) will be signed prior to enrollment.

### 2.4 Randomization, allocation, concealment and blinding

Randomization will be generated with the use of a random-number method via a computerized central randomization system. Eligible PD patients will be allocated in a 1:1:1 ratio to three groups: verum acupuncture, sham acupuncture, or waitlist control. After confirming eligibility, investigators will request group assignment via the centralized system, which automatically assigns and communicates the allocation to the investigator for intervention implementation. The randomization sequence will remain concealed on a secure server until completion of participant enrollment, data collection, and analysis to prevent selection bias.

To ensure methodological rigor, blinding will be implemented between the verum and sham acupuncture groups via a sham placebo acupuncture device (SPAD; Patent No. 2023215153288). The participants in both groups will receive treatments in private rooms to prevent cross-group communication. Outcome assessors, independent third-party personnel blinded to group allocations, will conduct all the clinical evaluations. A primary blinding code will be applied to the statistical database, ensuring that statisticians remain unaware of group assignments during analysis.

Owing to the inherent nature of acupuncture interventions, full blinding between acupuncture groups (verum/sham) and the wait-list control group is unattainable. However, stringent separation of study phases (recruitment, intervention, assessment) and adherence to predefined standard operating procedures (SOPs) minimize bias risks. In cases of serious adverse events (SAEs) requiring urgent unblinding (e.g., life-threatening complications), the principal investigator will access the allocation code via a sealed envelope stored in a secure location, document the reason, and report to the ethics committee within 24 h. Post-trial, the success of participant blinding will be evaluated via structured questionnaires. If blinding is compromised, we will perform sensitivity analyses by including blinding success as a covariate in the primary outcome models to assess its impact on treatment effect estimates.

### 2.5 Intervention

All PD participants will receive eight sessions (twice weekly for 4 weeks) of either verum or sham acupuncture. The regimen was derived from our clinical practice and informed by a pilot study that demonstrated significant clinical improvements, with due consideration of the need to balance treatment intensity and participant burden. The wait-list control group will receive no acupuncture during the observation period but will be offered the intervention post-trial. Patients on antiparkinsonian medications (e.g., levodopa) will maintain their regimen, with levodopa equivalent doses (LED) calculated at baseline. During the trial, medication adjustments are strongly discouraged; however, if any adjustment becomes clinically necessary, the change will be fully documented in case report forms (CRFs). HCs will undergo baseline MRI only, without interventions.

**Figure 2:**
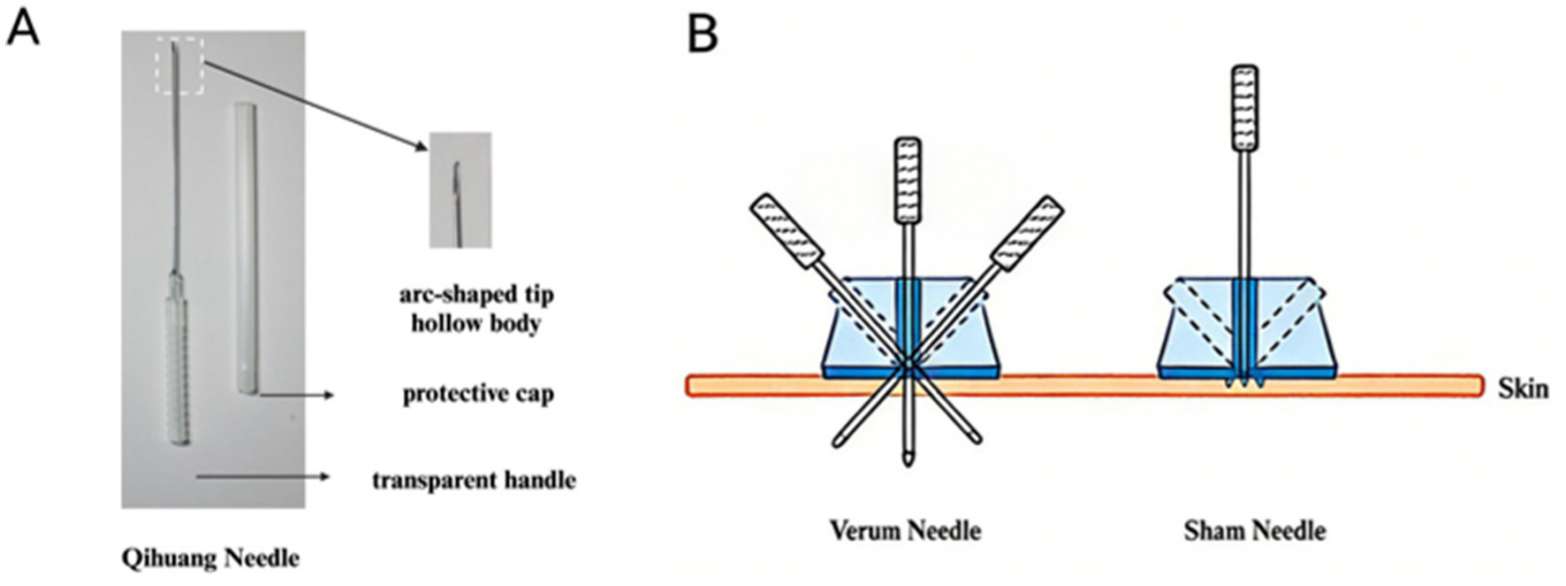
Qihuang needle and blind operation. **(A)** Qihuang needle, comprises four components: tip, body, handle, and protective cap. **(B)** A sham placebo acupuncture device (SPAD) integrates verum and sham needles. During application, the SPAD is attached to acupoints. For verum acupuncture, sharp needles penetrate the skin through the SPAD base. In contrast, sham needles feature a retractable blunt tip; upon contact with the base, internal microneedles within the SPAD simulate tactile penetration without skin puncture.

The verum and sham acupuncture groups will utilize the SPAD to ensure participant blinding. After standard skin disinfection, the SPAD is affixed to the target acupoint. For verum acupuncture, a sharp needle is inserted through the SPAD base to penetrate the skin. For sham acupuncture, a blunt-tip needle retracts upon contact with the skin, whereas microneedles within the SPAD simulate tactile penetration, replicating the deqi sensation without actual insertion. Figure 2 illustrates the blinding setup.

#### 2.5.1 Verum acupuncture group

The QNT employs disposable sterile acupuncture needles (Approval: 20202200072; 0.5 × 50 mm; Figure 2A) with a unique design: a hollow body, transparent handle, and arc-shaped tip. This configuration enhances mechanical strength and maneuverability, facilitating precise needle manipulation, robust deqi (sensation of soreness, numbness, or distension), reduced pain, and minimal vascular injury. A protective cap is used during acupoint transitions to prevent accidental needle-tip exposure.

**Figure 3:**
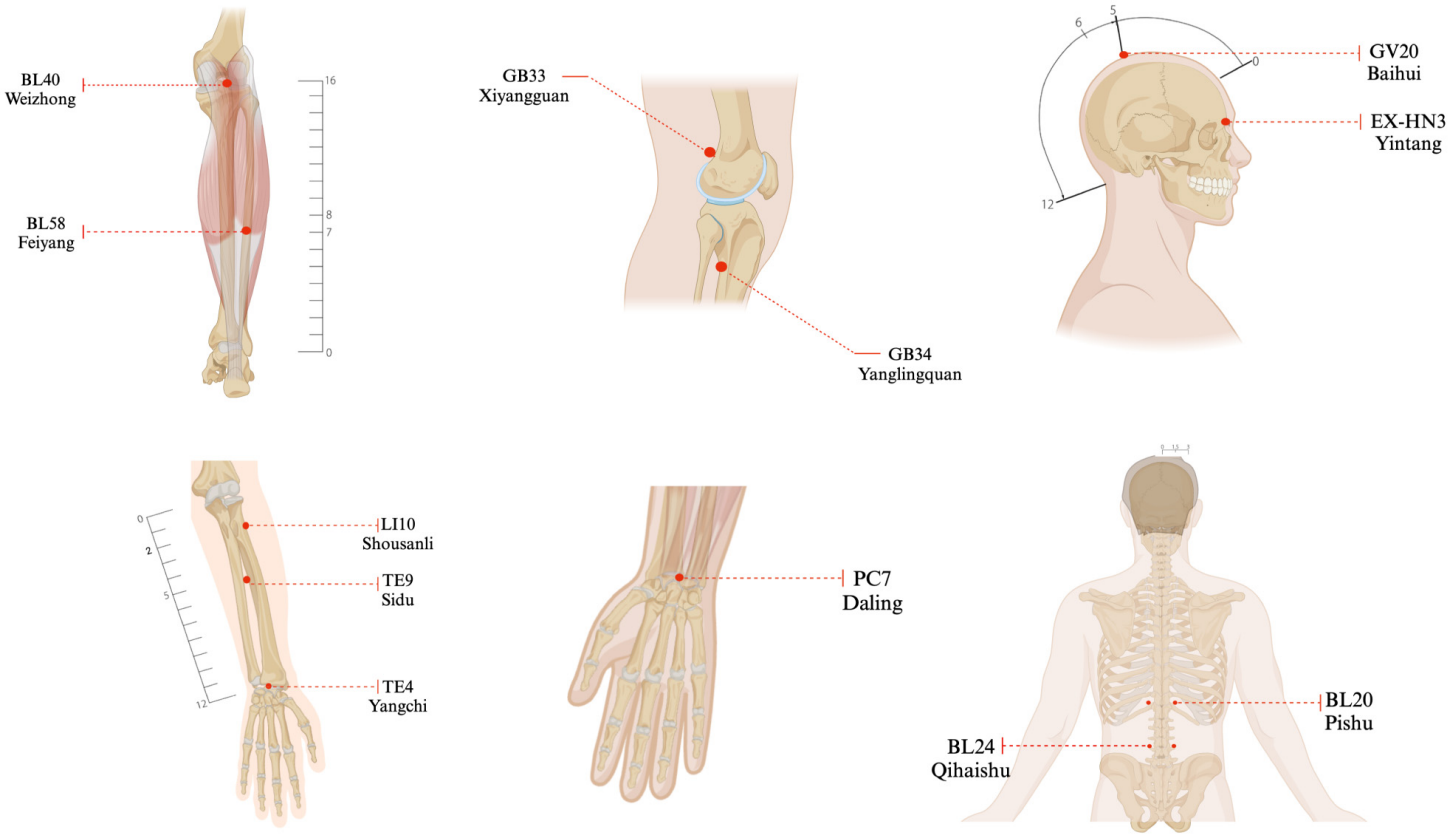
Location of acupoints.

On the basis of meridian-muscle theory and clinical expertise, participants in the verum acupuncture group will receive two weekly sessions over 4 weeks (8 sessions total) with the following protocol: acupoint prescription 1 (bilateral): EX-HN3 (Yintang), LI10 (Shousanli), TE4 (Yangchi), BL24 (Qihaishu), GB33 (Xiyangguan), and BL58 (Feiyang). Acupoint prescription 2 (bilateral): GV20 (Baihui), TE9 (Sidu), PC7 (Daling), BL20 (Pishu), BL40 (Weizhong), and GB34 (Yanglingquan). This sequence will be repeated weekly for 4 weeks. Acupoint localization follows the WHO Standard Acupuncture Point Locations in the Western Pacific Region (19) (Table 2, Figure 3).

**Table 2:** Location of acupoints.

Acupoint	Location
Acupoint prescription 1	
EX-HN3 (Yintang)	On the head, between the right medial end of the eyebrow and the left one.
LI10 (Shousanli)	On the posterolateral aspect of the forearm, on the line connecting LI5 with LI11, 2 Bcun inferior to the cubital crease.
TE4 (Yangchi)	On the posterior aspect of the wrist, in the depression ulnar to the extensor digitorum tendon, on the dorsal wrist crease.
BL24 (Qihaishu)	In the lumbar region, at the same level as the inferior border of the spinous process of the third lumbar vertebra (L3), 1.5 B-cun lateral to the posterior median line.
GB33 (Xiyangguan)	On the lateral aspect of the knee, in the depression between the biceps femoris tendon and the iliotibial band, posterior and proximal to the lateral epicondyle of the femur.
15.6-7.4,-38.3241ptBL58 (Feiyang)	On the posterolateral aspect of the leg, between the inferior border of the lateral head of the gastrocnemius muscle and the calcaneal tendon, at the same level as 7 Bcun proximal to BL60.
Acupoint prescription 2	
GV20 (Baihui)	On the head, 5 B-cun superior to the anterior hairline, on the anterior median line.
TE9 (Sidu)	On the posterior aspect of the forearm, midpoint of the interosseous space between the radius and the ulna, 5 B-cun distal to the prominence of the olecranon.
PC7 (Daling)	On the anterior aspect of the wrist, between the tendons of palmaris longus and the flexor carpi radialis, on the palmar wrist crease.
BL20 (Pishu)	In the upper back region, at the same level as the inferior border of the spinous process of the 11th thoracic vertebra (T11), 1.5 B-cun lateral to the posterior median line.
BL40 (Weizhong)	On the posterior aspect of the knee, at the midpoint of the popliteal crease.
GB34 (Yanglingquan)	On the fibular aspect of the leg, in the depression anterior and distal to the head of the fibula.

After standard skin disinfection, needles are swiftly inserted subcutaneously via the SPAD. Body and limb acupoints are inserted perpendicularly to a depth of 10–15 mm, whereas cranial acupoints undergo subcutaneous insertion at a depth of 5–10 mm. Upon achieving deqi, the needle is withdrawn to the entry point, followed by a Hegu manipulation (30–45 ° oblique redirection in opposing planes; Figure 2B) before removal.

#### 2.5.2 Sham acupuncture group

The participants will undergo procedures identical to those of the verum group, including acupoint selection and SPAD application, but non-penetrating blunt-tip needles will be used. The sham needles mimicked tactile stimulation without skin penetration, ensuring equivalent sensory feedback. Posttrial, the sham group participants will receive eight complimentary verum acupuncture sessions.

#### 2.5.3 Wait-list control group

During the 8-week observation period, wait-list controls will receive no acupuncture. To meet ethical standards, they will be offered eight free verum acupuncture sessions post-study, identical to the verum group's protocol.

### 2.6 Outcome measures

#### 2.6.1 Clinical outcomes

This trial is primarily designed to detect neuroplastic changes via multimodal MRI. Accordingly, the primary endpoint is the change in multimodal MRI biomarkers, whereas all clinical outcomes and biomechanical indices are designated as exploratory endpoints.

##### 2.6.1.1 Unified Parkinson's Disease Rating Scale part III (UPDRS-III)

Although all clinical analyses are exploratory, the change in UPDRS-III total score from baseline to week 4 is designated as the primary clinical outcome measure to maintain temporal alignment with the primary neuroimaging assessments. The UPDRS, revised by the Movement Disorder Society (MDS) in 2008 (20), is the gold standard for comprehensive PD symptom assessment (21). The Chinese version of the PD scale has excellent reliability and validity in Chinese PD patients (22). The UPDRS-III evaluates motor function via 18 items scored from 0–4 (higher scores indicate greater impairment). Assessments will occur at baseline, week 4 (post-treatment), and week 8 (end of follow-up; Table 1).

##### 2.6.1.2 Parkinson's disease non-motor symptom scale (NMSS)

Non-motor symptoms (NMS) often precede motor manifestations in PD patients and significantly impact disease progression and quality of life (23). The NMSS, developed and validated by the MDS in 2015, is a multidimensional tool that assesses NMS severity and frequency across 52 items within 13 domains (e.g., sleep, mood, gastrointestinal function) on a 0–4 scale. Higher total scores indicate greater non-motor symptom burden (24). NMSS assessments will be conducted at baseline, post-treatment (week 4), and at the end of follow-up (week 8).

##### 2.6.1.3 39-item Parkinson's disease questionnaire (PDQ-39)

The quality of life of PD patients is generally poorer than that of healthy individuals, particularly in terms of physical functioning and mental health (25). The PDQ-39 is a validated patient-reported outcome measure evaluating quality of life in PD across eight domains: mobility, activities of daily living, emotional well-being, stigma, social support, cognition, communication, and bodily discomfort (26). Each of the 39 items is scored 0–4 (higher scores = greater impairment), with total scores transformed to a 0–100 scale for interpretation. PDQ-39 assessments will be performed at baseline, week 4, and week 8.

##### 2.6.1.4 Biomechanical Index

Early-stage PD is characterized by reduced arm swing and shortened stride length (27), whereas advanced-stage PD is characterized by impaired balance, postural instability, muscle rigidity, and increased fall risk (28). Pathophysiological overlaps between PD and dystonia further underscore the relevance of biomechanical assessments (29). The FootScan system can be used to quantify gait parameters (step symmetry, center-of-pressure trajectory, temporal/spatial metrics), whereas shear wave elastography (SWE) can be used to evaluate muscle tone improvements. Biomechanical data will be collected at baseline and week 4.

#### 2.6.2 Multimodal MRI data

Multimodal MRI data will be acquired at baseline and post-treatment (week 4) via a Siemens Prisma 3.0T scanner (64-channel head coil). All scans will be performed between 4:00–6:00 p.m. Participants will be scanned in the practical “ON” medication state, approximately 1 h after their regular afternoon dopaminergic medication to maintain a uniform medication condition and minimize motion artifacts caused by motor symptoms. No specific dietary restrictions are required before scanning. The post-treatment scan will be scheduled within 24 h after the final acupuncture session. Structural and functional imaging protocols include the following structural markers: T1-weighted imaging (T1WI), T2-weighted imaging (T2WI), T2-Flair imaging, diffusion tensor imaging (DTI), neuromelanin-sensitive MRI (NM-MRI), and susceptibility-weighted imaging (SWI). Functional markers: blood oxygen level-dependent functional MRI (BOLD-fMRI). Detailed acquisition parameters, quality control procedures, preprocessing, and statistical thresholds are provided in Supplementary material 3.

##### 2.6.2.1 Structural MRI (sMRI) and voxel-based morphometry (VBM)

PD is associated with widespread cortical gray matter atrophy (e.g., frontal lobe, cingulate cortex), where reduced gray matter volume is correlated with cognitive decline (30). sMRI involves automated whole-brain analysis to assess the characteristics of gray matter, white matter, and cerebrospinal fluid. VBM, as a method of sMRI image processing, aids in differential diagnosis, and monitoring disease progression (31). The imaging parameters were standardized as follows: T1WI will be acquired using a 3D MPRAGE sequence (TR/TE/TI = 2,300/2.98/900 ms, flip angle = 9°, voxel size = 1 × 1 × 1 mm3, 176 sagittal slices, acquisition time approximately 5–6 min). T2WI will be acquired using a 2D turbo spin-echo sequence (TR/TE = 3,650/92 ms, slice thickness/gap = 5/1.5 mm). T2-FLAIR will be acquired using a 2D sequence (TR/TE/TI = 9,000/120/2,500 ms, slice thickness/gap = 5/1.5 mm).

##### 2.6.2.1 Resting-state fMRI (rs-fMRI)

rs-fMRI leverages blood oxygen level-dependent (BOLD) signals to map spontaneous neural activity and functional connectivity, capturing neurovascular uncoupling patterns in PD (32). Aberrant connectivity within the basal ganglia-thalamocortical circuit and dysregulation of posterior cortical regions, the caudate, and the cerebellum have been mechanistically linked to both motor and non-motor symptoms (33). These insights advance the understanding of PD pathophysiology and inform therapeutic development. Imaging Protocol: simultaneous multislice (SMS) BOLD sequence (multiband factor = 4): TR/TE = 500/30 ms, field of view = 224 × 224 mm^2^, voxel size = 3.5 × 3.5 × 3.5 mm3, 35 axial slices, 960 volumes, acquisition time = 8 min. Participants will be instructed to keep their eyes closed, remain still, and stay awake.

##### 2.6.2.3 Diffusion tensor imaging (DTI)

DTI quantifies microstructural integrity and evaluates the microstructural integrity of nigrostriatal pathways through water diffusion anisotropy (34). Reduced fractional anisotropy (FA) in the substantia nigra, putamen, and caudate reflects dopaminergic degeneration, serving as an early biomarker for connectivity disruption and disease progression (35). Imaging Protocol: SMS-DTI sequence: TR/TE = 4,200/72 ms, ST/SG = 2/1 mm, voxel size = 2 mm3, 99 diffusion directions, b-values = 0–3,000 s/mm^2^ (gradient distributions: 0, 300, 350, 650, 950, 1,000, 1,350, 1,650, 1,700, 2,000, 2,700, 3,000).

##### 2.6.2.4 Susceptibility-Weighted Imaging (SWI) and Neuromelanin MRI (NM-MRI)

In PD, iron accumulation in nigrosome-1 and neuromelanin depletion in the substantia nigra pars compacta (SNc) are pathognomonic (36). SWI detects iron deposition via phase-sensitive contrast (37), whereas NM-MRI tracks neuromelanin loss through T1-shortening effects (38), offering high diagnostic specificity for early PD detection. Imaging protocols: SWI will be acquired using a 3D flow-compensated gradient-echo (GRE) sequence, saving both magnitude and phase images (TR/TE = 27/20 ms, flip angle = 15°, voxel size = 1 × 1 × 1 mm3, slice thickness = 1 mm, acquisition time ≈ 5–7 min). NM-MRI will be acquired using a T1-weighted turbo spin-echo sequence (TR/TE = 600/13 ms, slice thickness = 2.5 mm, field of view = 220 × 220 mm^2^, acquisition time ≈ 8 min).

#### 2.6.3 Others

##### 2.6.3.1 Medication monitoring

During the observation period, participants will maintain a standardized PD diary to document all concomitant medications and healthcare resource utilization aimed at managing PD symptoms. Diary entries will be cross-verified with electronic medical records during follow-up visits to ensure accuracy and completeness.

##### 2.6.3.2 Acupuncture expectancy assessment

Prior to the first intervention, participants will complete the validated Patients' Expectancy Scale of Acupuncture (PESA) (39), an 11-item instrument evaluating four domains: disease-related expectations, treatment-related beliefs, process-related concerns, and outcome-related projections. Responses are recorded on a 10-point Likert scale (1 = strongly disagree to 10 = strongly agree). Total scores will be analyzed as covariates to assess expectancy effects on therapeutic outcomes.

##### 2.6.3.3 Blinding assessment

To evaluate blinding success, participants in the verum and sham acupuncture groups will complete a post-intervention questionnaire after their first, fourth, and final sessions. The assessment comprises the following: (1) treatment type guess: “Which intervention do you believe you received?” (Options: traditional acupuncture, acupuncture-like stimulation, uncertain). (2) Treatment preference confidence: a 10-cm visual analog scale (VAS) anchored by sham acupuncture (0 cm) and verum acupuncture (10 cm) was used.

##### 2.6.3.3 Deqi sensation assessment

To objectively assess deqi sensation, patients will rate its intensity immediately after each session on a 10-cm visual analog scale (VAS), anchored with 0 = “no sensation” and 10 = “unbearable sensation”.

##### 2.6.3.4 Patient compliance assessment

Patient compliance will be recorded at the end of the treatment. Dropouts and reasons will be documented during the 8-week observation period.

### 2.7 Safety assessments

All adverse events (AEs), including symptom severity, onset time, interventions, outcomes, and follow-up details, will be documented in CRFs. For SAEs leading to participant withdrawal, a dedicated withdrawal form will record the reason, last treatment date, completed assessments, and SAE resolution status. All SAEs will be reported promptly to the institutional review board, principal investigator, and trial coordinator. AE causality will be assessed, and all events will be monitored until resolution or stabilization. Reporting will adhere to CONSORT guidelines for non-pharmacological trials.

### 2.8 Data management and quality control

Prior to study initiation, all personnel will undergo standardized training on protocol adherence, outcome measurement protocols, and eligibility criteria. Clinical data will be systematically recorded in CRFs with built-in validation checks and stored securely. Data access will be restricted to authorized personnel until study completion to prevent premature analysis bias. The quality assurance (QA) team will perform bimonthly audits, evaluating protocol compliance, data accuracy, and procedural consistency. Licensed acupuncturists must hold ≥2 years of specialized training in the QNT, verified through competency assessments. To ensure consistency of the acupuncture technique across practitioners, all acupuncturists will undergo a standardized training session and pass a practical examination before the trial commences. During the trial, procedural checklists will be used for each treatment session, and video recordings of a random 10% of sessions will be reviewed by an independent expert to verify adherence to the standardized manipulation protocol. Any deviations will be discussed in regular team meetings to maintain treatment fidelity. All scans will be acquired on a single Siemens Prisma 3.0T scanner (64-channel head coil) at the First Affiliated Hospital of Guangzhou University of Chinese Medicine, operated by two certified MRI technicians.

An independent Data Monitoring Committee (DMC) comprising a neurologist, a statistician, and an acupuncture therapy expert will be established. The DMC will review interim safety data every three months, assess trial conduct, and recommend continuation or termination on the basis of predefined stopping rules (e.g., SAE rate exceeding 10%). The DMC operates independently from the sponsor and investigators, with all communications documented in meeting minutes. As this is a mechanistic trial with neuroimaging as the primary endpoint, no interim efficacy or futility analysis is planned, as early termination for efficacy would preclude adequate collection of neuroplasticity data.

### 2.9 Statistical analysis

#### 2.9.1 Clinical data analysis

All statistical tests will be two-tailed, with significance set at α = 0.05. Baseline demographic and clinical characteristics will be summarized descriptively by group. Baseline demographic and clinical characteristics will be summarized descriptively by group. To assess baseline comparability among the three randomized groups, one-way ANOVA (or Kruskal–Wallis test) will be used for continuous variables, and chi-square tests for categorical variables. If significant between-group differences are observed at baseline, the affected variables will be included as covariates in subsequent analyses to adjust for potential confounding. For the primary clinical outcome, no correction for multiple comparisons is applied as this is a single predefined comparison. For secondary clinical outcomes, we will apply False Discovery Rate (FDR) correction within each assessment scale. Continuous data will be analyzed via one-way ANOVA (normally distributed data) or the Kruskal–Wallis *H*-test (non-normal data), with Bonferroni-adjusted *post-hoc* comparisons. Categorical variables will be analyzed using the Pearson χ^2^ test or Fisher's exact test for unordered variables, and the linear trend χ^2^ test for ordered variables. Analysis of covariance (ANCOVA) will be employed to adjust for baseline values, age, disease duration, and levodopa equivalent daily dose where appropriate. If significant baseline differences between groups are observed after randomization, these variables will be included as covariates in the corresponding models to mitigate bias. Missing data will be handled using multiple imputation by chained equations under the missing at random assumption, with sensitivity analyses comparing imputed results to complete-case analyses. An intention-to-treat (ITT) analysis will include all randomized participants, whereas per-protocol analyses will exclude non-compliant cases (< 80% session attendance).

#### 2.9.2 Neuroimaging data analysis

Neuroimaging data will be analyzed according to standardized pipelines, with comprehensive details provided in Supplementary material 3. For VBM, the CAT12 toolbox (SPM12, version r1720; CAT12, r1720) will be used for bias correction, tissue segmentation, and spatial normalization to MNI space. Whole-brain VBM results will be reported at cluster-level family-wise error (FWE) corrected *p* < 0.05 with an initial voxel-level threshold of *p* < 0.001. Resting-state fMRI data will be preprocessed using DPABI v6.1. Preprocessing will include discarding the first 10 volumes, slice timing correction, realignment, normalization to MNI space, spatial smoothing (6-mm FWHM), nuisance regression (Friston-24 motion parameters and WM/CSF signals), detrending, and bandpass filtering (0.01–0.08 Hz). Volumes with framewise displacement (FD) > 0.5 mm will be censored using scrubbing regressors; participants with >20% censored volumes or with maximum head motion >3.0 mm translation or 3.0° rotation will be excluded. Seed-based functional connectivity will be computed using predefined seeds from the Automated Anatomical Labeling (AAL) atlas (bilateral putamen, caudate, globus pallidus, and thalamus; seed coordinates are provided in Supplementary material 3). Voxel-wise connectivity maps (Fisher's z-transformed) will be assessed using cluster-level FWE correction (voxel *p* < 0.001; cluster *p* < 0.05). To address multiple comparisons arising from testing multiple seeds, seed-level multiplicity will be controlled using false discovery rate (FDR; Benjamini–Hochberg) across seeds (*q* < 0.05). In addition, the bilateral putamen will be designated *a priori* as the primary seed of interest, whereas results from the remaining seeds will be considered secondary/exploratory and interpreted with FDR control across seeds. DTI data will be processed using DTIStudio v3.0.3 (and the NODDI toolbox in MATLAB R2013b, if applicable). Fractional anisotropy (FA), mean diffusivity (MD), radial diffusivity (RD), and axial diffusivity (AD) will be computed. Regions of interest (substantia nigra, putamen, caudate) will be manually delineated on FA color maps by two independent raters blinded to group and time point, with inter-rater reliability assessed using intraclass correlation coefficients (ICC; ICC > 0.80 required). ROI-based statistical comparisons will apply Bonferroni correction within each predefined ROI family. SWI phase images will undergo high-pass filtering (32 × 32) before manual delineation of substantia nigra (SN) area and measurement of SN pars compacta (SNc) width. NM-MRI SNc width will be measured in the dorsoventral direction on the optimal axial slice using the ADW4.6 workstation. ICC will be assessed for all manual measurements, and the average of the two raters will be used for analysis. All statistical tests will be two-tailed with significance set at *p* < 0.05 after correction.

### 2.10 Ethics and dissemination

This trial complies with the Declaration of Helsinki and is registered at the International Traditional Medicine Clinical Trial Registry (No. ITMCTR2025000402). Written informed consent (Supplementary material 2) will be obtained after full disclosure of the study objectives, procedures, and risks. Participant confidentiality will be strictly maintained through anonymized data coding. Findings will be disseminated via peer-reviewed publications and conference presentations. Deidentified data will be shared upon reasonable request 3 years post-publication, contingent on data use agreements and institutional approval.

## 3 Discussion

This triple-arm RCT integrates multimodal MRI to evaluate QNT's efficacy and neuroplastic mechanisms in PD. The three-arm design (verum, sham, wait-list) is essential to disentangle specific effects from placebo and disease progression. Although an 8-week wait-list period may raise ethical considerations, all participants continue standard anti-parkinsonian medication, ensuring basic care is maintained. To our knowledge, this is the first study to systematically apply a comprehensive neuroimaging battery encompassing resting-state functional connectivity, gray matter volumetry, white matter integrity, nigral iron, and neuromelanin to elucidate acupuncture's neuroplastic mechanisms in PD, providing a novel methodological framework for mechanistic validation of traditional therapies.

Acupuncture has gained global recognition as a safe, cost-effective, and accessible adjunct therapy for PD (40). Guided by the principles of *Huangdi Neijing*, the QNT integrates “nine needle theory” and “five needling techniques” with viscera-meridian syndrome differentiation to achieve precision treatment. Its disposable sterile needles (Approval: 20202200072) feature a transparent handle, arc-shaped tip, and hollow body, combined with unique acupuncture techniques, enabling stronger deqi sensation, minimal discomfort, and shorter session duration. By targeting PD hallmark motor dysfunction, the protocol prioritizes acupoints guided by meridians-muscles theory, which has been shown to effectively alleviate joint pain and functional activity disorders (41). BL24 and BL32 enhance lumbosacral qi infusion; address postural instability; LI10, TE4, TE9, and PC7, which are located near the elbow and wrist joints, can help unblock the meridians and improve symptoms in the upper limb. Acupuncture at GB33, BL58, BL40, and GB34, which are situated near lower limb joints and tendons, is particularly suitable for treating lower limb dysfunctions such as difficulty initiating gait and rigidity. Additionally, EX-HN3 and GV20 are head points that stimulate local qi and blood circulation and promote neuroplasticity. Research has shown that acupuncture at EX-HN3 and GV20 can increase cerebral blood flow and inhibit neuroinflammation (42, 43), thereby facilitating neuroprotection. Clinical observations support the efficacy of the QNT's in ameliorating motor/non-motor symptoms and quality of life in PD[6], yet its mechanistic underpinnings remain underexplored.

Building on emerging evidence supporting acupuncture's neuromodulatory effects, multimodal neuroimaging provides a robust framework to systematically decode its mechanisms. Evidence suggests it may modulate basal ganglia-thalamocortical circuits (44), increasing regional blood flow (45) and activating cortical metabolic pathways (46) supporting its neuromodulatory effects. Preclinical studies have demonstrated that acupuncture at ST36 (Zusanli) reduces basal ganglia, limbic, and cerebellar BOLD signals in PD models (47), whereas human trials have revealed enhanced neural responses in the substantia nigra, caudate, thalamus, and putamen following GB34 (Yanglingquan) stimulation (48). We hypothesize that QNT may promote the functional reorganization of the basal ganglia-thalamocortical circuits and associated motor networks through stimulation of acupoints, and may ameliorate pathological iron deposition in the substantia nigra, potentially via the modulation of these neural circuits. If the QNT demonstrates positive effects on neuroplasticity and symptom alleviation, this study will provide high-level mechanistic evidence supporting the integration of acupuncture into standardized neurorehabilitation protocols for PD.

This trial has several strengths: first is intervention optimization, minimal number of acupoints, no needle retention, rapid efficacy, and high safety increase patient compliance and reduce needle phobia. Second, multimodal MRI technology will be utilized to quantitatively assess gray matter volumetry (VBM), analyze default mode-sensorimotor network connectivity (rs-fMRI), evaluate white matter integrity (DTI), and quantify iron deposition (SWI) and neuromelanin signal intensity (NM-MRI) to map neuroplasticity targets. Third, the integration of clinical scales, biomechanical indices and neuroimaging biomarkers provides robust mechanistic validation. Fourth, triple-arm randomization (verum acupuncture, sham acupuncture, waitlist control) with strict allocation concealment, SPAD blinding protocols, and post-trial blinding assessment ensures internal validity.

Several constraints need to be acknowledged. First, practitioner/patient blinding is infeasible between the verum and waitlist groups, which may introduce performance and detection bias. Second, the sham acupuncture group received non-penetrating stimulation at the same acupoints as the verum group, which is essential for maintaining participant blinding via the SPAD device. However, superficial needling at true acupoints may exert mild physiological effects (49), potentially diluting between-group differences. The inclusion of a wait-list control group helps to estimate and partially disentangle these non-specific effects. Third, the relatively short follow-up period, while sufficient to detect short-term neuroplastic changes, is inadequate for assessing the long-term durability of treatment effects. Future research should extend the follow-up duration (e.g., 12-24 months) to determine whether QNT-induced neuroplasticity translates into sustained clinical benefits. Fourth, this study is conducted at a single center, whereas ensuring procedural consistency, multicenter replication is essential for generalizability. Furthermore, whereas MRI provides excellent spatial resolution for structural and functional connectivity, it lacks the temporal resolution to capture dynamic neural oscillations in real-time. Future studies could integrate electroencephalography (EEG) to complement MRI findings (50, 51), offering a more comprehensive view of brain network dynamics during and after acupuncture intervention.

This study pioneers the synergistic application of multimodal MRI (structural, functional, and neuromelanin-sensitive imaging) within a triple-arm RCT framework. By correlating clinical outcomes (e.g., UPDRS-III, NMSS) with neuroimaging biomarkers (e.g., nigral iron deposition, functional connectivity), we aim to establish causal links between the QNT-induced neuroplasticity and symptom alleviation, offering a paradigm for mechanistic validation of traditional therapies and informing health policy decisions regarding the inclusion of TCM in multidisciplinary PD care pathways.
