# Supplementary material for: Qihuang needle therapy for Parkinson's disease: a triple-arm randomized controlled trial protocol assessing efficacy and neuroplasticity via multimodal MRI
Source: Front Neurol. 2026 Apr 13;17:1722126. doi: 10.3389/fneur.2026.1722126 (PMC13112538; doi:10.3389/fneur.2026.1722126)
Supplement: Supplementary file 2 [file Data_Sheet_2.pdf]

Clinical Research Informed Consent Form (for Investigational Studies)

Project Title: Prospective Randomized Controlled Clinical Study Protocol for the Therapeutic Efficacy of Acupuncture in Parkinson's Disease
Sponsor (Applying Department): Acupuncture and Rehabilitation Center
Clinical Trial Institution: The First Affiliated Hospital of Guangzhou University of Chinese Medicine
Version Number or Date: V4.0 or January 6, 2025
Principal Investigator (only fill in one person): Nanbu Wang

Personal Reading Material

Dear Participant,

Please read this document carefully and feel free to ask questions and discuss it with your family, relatives, friends, or us.。

You are being invited to participate in this clinical research. The purpose of the study is to explore the clinical efficacy of acupuncture treatment in patients with Parkinson's disease (PD).

Participation in this study is entirely at your discretion. Please read this material thoroughly before making a decision. It will help you fully understand the objectives, methods, study process, potential benefits and inconveniences, and your rights in this study. The information provided in this informed consent form can help you decide whether to participate in this clinical trial. If you have any questions, please ask the researcher responsible for the project, or discuss it with your family, relatives, or friends to ensure you fully understand the content. Your participation in this trial is voluntary. If you agree to participate in the clinical trial, please sign the declaration section of the informed consent form.

I. What Kind of Study is This? Study Background and Objectives

This study is titled "Prospective Randomized Controlled Clinical Study Protocol for the Therapeutic Efficacy of Acupuncture in Parkinson's Disease." This study has been reviewed and approved by the Ethics Committee of the First Affiliated Hospital of Guangzhou University of Chinese Medicine, which considers that this study complies with the internationally recognized principles of the Declaration of Helsinki and meets medical ethics.

(A) Current Treatment Methods for Parkinson's Disease

1. Pharmacotherapy: Levodopa preparations, non-ergot dopamine agonists, monoamine oxidase B (MAO-B) inhibitors, catechol-O-methyltransferase (COMT) inhibitors, anticholinergic drugs, etc.
2. Acupuncture Therapy: Acupuncture can dredge the meridians, circulate qi and blood, connect

organs, and restore the balance of yin and yang in the body. It is a safe, effective, and convenient method widely used in the clinical treatment of Parkinson's disease.

3. Surgical Treatment: Botulinum toxin injection therapy, deep brain stimulation (DBS), repetitive transcranial magnetic stimulation (rTMS)

4. Exercise and Rehabilitation Therapy: Language and swallowing function training, gait training, posture balance training, as well as Tai Chi, yoga, dance, hydrotherapy, meditation, etc., which help improve the motor and non-motor symptoms of patients with Parkinson's disease.

5. Care and Psychological Counseling: Provide disease education and psychological counseling for patients and their families, increase confidence in treatment and rehabilitation, help improve depression, anxiety, and other emotions, and at the same time, scientifically care for and increase nutrition, pay attention to preventing aspiration and falls and other accidents will have a positive auxiliary therapeutic effect.

(B) Introduction to the Methodology of This Study

The objective of this study is to explore the clinical efficacy of acupuncture treatment in patients with Parkinson's disease.

This study will be conducted at the First Affiliated Hospital of Guangzhou University of Chinese Medicine, and it is expected to recruit approximately 165 patients. If you agree to participate in this clinical study, you will be randomly assigned to one of the following groups: acupuncture group (basic drug treatment + acupuncture treatment); sham acupuncture group (basic drug treatment + sham acupuncture treatment); or waiting group (basic drug treatment only, with 8 free acupuncture treatments offered after the 24-week observation period).

The leading undertaking unit of this study is the First Affiliated Hospital of Guangzhou University of Chinese Medicine, which has been approved by the Ethics Committee of Guangzhou University of Chinese Medicine. The Ethics Committee has reviewed and approved this study as complying with the principles of the Declaration of Helsinki and meeting medical ethics.

II. Is Participation in This Study Voluntary?

Participation in this study is voluntary. You have the right to decide whether to participate in this study, and there is no need for any reason to not participate. Not participating in this study will not result in any discrimination or retaliation, will not affect your relationship with the doctor, nor will it affect your medical benefits. You will continue to receive medical treatment from your doctor.

III. Who Should Not Participate in This Study?

If you have any of the following conditions, you will not be suitable for this study: (1) Parkinsonism caused by various causes such as cerebrovascular disease, poisoning, etc., progressive supranuclear palsy, multiple system atrophy, Lewy body dementia, and corticobasal degeneration; (2) Those with serious diseases such as severe liver and kidney, blood, tumors, endocrine diseases, or pain disorders; (3) Those participating in other drug and acupuncture clinical trials; (4) Those with mental illness; (5) Those with deafness or communication barriers; (6) Those with a history of alcoholism or drug abuse; (7) Pregnant women or those planning to become pregnant.

IV. What Will Be Required If You Participate in the Study?

Before you are selected for the study, you will undergo the following examinations to determine if you are eligible to participate in the study.

The doctor will inquire about and record your medical history, conduct a comprehensive physical examination, and perform relevant rehabilitation assessments.

If you meet all the following conditions, you may be eligible to participate in this study:

1. Meet the diagnostic criteria for Parkinson's disease according to Western medicine

(1) Refer to the "Chinese Diagnostic Criteria for Parkinson's Disease (2016 Edition)"

(2) Core symptoms of Parkinson's disease: ① Bradykinesia. ② Rigidity and/or resting tremor (4-6Hz).

(3) Must meet 2 or more supportive criteria for diagnosing Parkinson's disease, such as:

·Significant and clear effectiveness of dopaminergic treatment.

·Presence of levodopa-induced dyskinesia.

·Presence of resting tremor in a single limb in previous or current physical examination.

·Loss of smell, or head ultrasound showing high signal in the substantia nigra or cardiac sympathetic denervation indicated by MIBG scintigraphy.

2. Age 25-80 years;

3. Hoehn-Yahr stage 1-4;

4. Conscious, stable vital signs, without significant intellectual disability, hearing without significant impairment;

5. Signed informed consent form.

V. Steps to Follow If You Qualify

The study will randomly determine whether you receive acupuncture, sham acupuncture treatment, or no treatment. Patients participating in the study have a 1/3 chance of being assigned to these three different groups. Neither you nor your doctor can know or choose any treatment method in advance. The treatment observation will last for 24 weeks.

All enrolled patients will continue to maintain oral basic drug treatment.

Acupuncture Group: Basic drug treatment + acupuncture treatment. Disposable sterile acupuncture needles (Approval No.:渝械注准:20202200072) will be used. The treatment cycle is 4 weeks, twice a week, totaling 8 times.

Sham Acupuncture Group: Basic drug treatment + sham acupuncture treatment. It can simulate the feeling of acupuncture treatment without actually piercing the skin, the treatment cycle is 4 weeks, twice a week, totaling 8 times. After the 24-week observation period, 8 free acupuncture treatments will be offered, with the same treatment plan as acupuncture.

Waiting Group: Basic drug treatment only. After the 24-week observation period, 8 free acupuncture treatments will be offered, with the same treatment plan as acupuncture.

Assessment at 4, 8, 12, and 24 weeks: You should visit the hospital for treatment, and the doctor will collect your medical history and perform relevant rehabilitation assessments again. Please honestly reflect any changes in your condition to the doctor, who will record the changes in your condition and perform a physical examination and related biochemical tests. At the same time, we will collect your peripheral venous blood before and at 4 and 24 weeks of treatment to collect your response to acupuncture therapy and your health status, which will not affect your clinical diagnosis and treatment process.

VI. Potential Benefits of Participating in the Study

You and society may benefit from this study, or there may be no direct benefit. Such benefits include the possibility of improvement in your condition, as well as this study may help develop new treatment and auxiliary diagnostic methods, further clarifying the clinical efficacy of acupuncture treatment in patients with Parkinson's disease, to be used for other patients with similar conditions.

VII. Potential Adverse Reactions, Risks, and Inconveniences of Participating in the Study

All treatment methods may have side effects. The acupuncture method used in this study may

cause fainting, stagnation of needles, local hematoma, etc., in some patients. Overall, the adverse reactions are relatively few. If you experience any discomfort or new changes in your condition during the study, such as increased tremors, or any accidents, regardless of whether they are related to the treatment method, you should notify your doctor in a timely manner. He/she will make a judgment and provide medical treatment, and the doctor will do his/her best to prevent and treat any harm that may arise from this study.

In addition, any treatment may be ineffective, and the condition may continue to progress due to ineffective treatment or due to the combination of other diseases. This is a treatment risk that every patient seeking medical treatment will face, and even if you do not participate in this clinical study, the treatment risk will exist. During the study period, if the doctor finds that the treatment measures taken in this study are ineffective, the study will be terminated, and other potentially effective treatment measures will be adopted.

You will need to keep appointments with the hospital during the study period, do some biochemical tests, which may cause trouble or inconvenience to you.

VIII. Costs

The research team will cover the costs related to your participation in this study, including: blood routine, urine routine, liver and kidney function, motor function instrument examination fees, imaging examination fees, and acupuncture treatment fees; rehabilitation training, and routine drug treatment costs will be charged according to medical standards.

If you have other diseases concurrently and unrelated to this study, such as hypertension, diabetes, coronary heart disease, etc., the treatment and examination costs for these basic diseases, as well as the costs for changing to other treatment measures due to the termination of the study, will not be covered for free. If any damage related to the study occurs, the research team will provide treatment costs for the damage related to the study and give corresponding economic compensation according to national laws and regulations.

IX. How to Protect Your Privacy Rights?

Your medical records (study medical records/CRF, biochemical examination reports, etc.) will be kept intact in the hospital according to regulations. The doctor will record the results of the rehabilitation assessment in your clinical case observation form. Your personal information and

medical information in this study are confidential. Any public reports of the results of this study will not disclose your personal identity. Third-party cooperative institutions involved in the testing will sign confidentiality agreements with us. Superior health/pharmaceutical/scientific research management departments, hospital ethics committees, researchers, and sponsors will be allowed to access your medical records to verify the procedures and/or data of the clinical study. By signing this informed consent form, you agree to use your personal and medical information for the purposes described above, and we will strictly protect the privacy of your personal medical information within the scope permitted by existing laws.

X. Important Reminders

1. Other Matters That Need Your Cooperation

To ensure the reliability of the clinical study, we hope you can do the following during the study process: ① You need to take medication as directed by the doctor, and please record it objectively in the "Medication Record Card" after each dose. During the study period, if you need to take other drugs or receive other treatments outside the study, please consult the study physician first; ② If you have any discomfort during the study process, please report it to your attending doctor in a timely manner. ③ You need to complete the systematic treatment and related examinations and assessments at the hospital according to the agreed time with your doctor. Your follow-up is very important because the doctor will judge whether the treatment you receive really works. ④ Regarding diet and lifestyle regulations: low-fat, low-salt diet, quit smoking, maintain emotional stability.

2. Expected Situations and/or Reasons for Possible Termination of Your Participation in the Trial

① Failure to strictly follow the treatment plan during the treatment period, such as the subject using other similar drugs (or treatments) on their own (or the researcher increasing or reducing acupoints on their own); ② The subject cannot be contacted due to address or phone number changes during the follow-up period, or does not cooperate with the follow-up observation, resulting in the inability to observe the efficacy on time.

The treatment plan of this study is not the only treatment option for your current disease. You can discuss with the doctor before deciding whether to participate in this study.

XI. Can You Withdraw from the Study After Participating?

Whether to participate is entirely up to your voluntary decision. You can refuse to participate in

this study, or withdraw from the study at any time without any reason, which will not affect your relationship with the doctor, nor will it affect the loss of your medical or other interests. You will not be discriminated against or retaliated against.

Your doctor may terminate your participation in this study at any time for the best interests of you.

If you do not participate in this study, or withdraw midway, there are other alternative treatment measures. You do not have to participate in this study just to treat your disease. If you withdraw from the study for any reason, for the best interests of you, you may be asked about your medication use, and if the doctor thinks it is necessary, you may also be required to undergo laboratory tests and physical examinations.

If you decide to participate in this study after careful consideration, we hope you can complete the entire study process.

XII. Access to More Information

You can ask any questions about this study at any time, and your doctor will leave contact information so that he/she can answer your questions.

If there is any important new information during the study process that may affect your willingness to continue participating in the study, your doctor will notify you in a timely manner.

XIII. What to Do Now?

Before you make a decision to participate in the study, please ask your doctor as many questions as possible until you fully understand the study.

Whether to participate in this study is up to you to decide. You can also discuss it with your family before making a decision.

Thank you for reading the above material. If you decide to participate in this study, please tell your doctor, and they will arrange everything related to the study for you.

Please keep this document.

If you have any questions about your rights in this study, you can contact the Ethics Committee of our center, telephone: 020-36588667; email: gztcmlunli@163.com.

Participant Declaration

I have carefully read this informed consent form, I had the opportunity to ask questions and all questions have been answered. I understand that participation in this study is voluntary, I can choose not to participate in this study, or notify the researcher at any time to withdraw without being discriminated against or retaliated against, my any medical treatment and rights will not be affected.

If I need other diagnosis/treatment, or I did not comply with the trial plan, or have other reasonable reasons, the researcher can terminate me from continuing to participate in this clinical study.

I voluntarily agree to participate in this clinical study, I will receive a signed original "informed consent form" (including personal reading material and participant declaration page).

Participant Signature: Date:

Contact Phone:

Legal Agent Signature [if applicable]: Contact Phone:

Relationship with Participant: Date:

Investigator Declaration

I have accurately informed the participant of the contents of the informed consent form and answered the participant's questions. The participant voluntarily participates in this clinical study.

Investigator Signature: Date:

Contact Phone:
