# Supplementary material for: Qihuang needle therapy for Parkinson's disease: a triple-arm randomized controlled trial protocol assessing efficacy and neuroplasticity via multimodal MRI
Source: Front Neurol. 2026 Apr 13;17:1722126. doi: 10.3389/fneur.2026.1722126 (PMC13112538; doi:10.3389/fneur.2026.1722126)
Supplement: Supplementary file 3 [file Data_Sheet_3.docx]

**Supplementary Material 3: Detailed Neuroimaging Protocol**

**1. MRI Acquisition Parameters**

All images will be acquired on a Siemens Prisma 3.0T scanner with a 64-channel head coil. The standardized protocol is as follows:

①T1-weighted imaging (T1WI): 3D MPRAGE sequence, TR/TE = 2500/24 ms, flip angle = 90°, slice thickness/gap = 5/1.5 mm, voxel size = 1×1×1 mm³, matrix = 256×256, 176 slices, acquisition time ≈ 5–6 min..

②T2-weighted imaging (T2WI): 2D turbo spin-echo sequence, TR/TE = 3650/92 ms, flip angle = 90°, slice thickness/gap = 5/1.5 mm, matrix = 256×256, 35 slices.

③T2-FLAIR: TR/TE = 9000/120 ms, flip angle = 90°, slice thickness/gap = 5/1.5 mm, matrix = 256×256, 35 slices.

④Resting-state fMRI (rs-fMRI): Simultaneous multislice (SMS) BOLD sequence with multiband factor 4: TR/TE = 500/30 ms, field of view = 224×224 mm², spatial resolution = 3.5×3.5×3.5 mm³, slice thickness = 3.5 mm, 35 axial slices, 960 volumes, acquisition time = 8 min. Participants will be instructed to keep eyes closed, remain still, and not fall asleep.

⑤Diffusion tensor imaging (DTI): SMS-DTI sequence with multiband factor 4: TR/TE = 4200/72 ms, slice thickness/gap = 2/1 mm, voxel size = 2×2×2 mm³, 99 diffusion directions, b-values = 0, 300, 350, 650, 950, 1000, 1350, 1650, 1700, 2000, 2700, 3000 s/mm², acquisition time = 12 min.

⑥Susceptibility-weighted imaging (SWI): 3D T1-weighted MPRAGE sequence with flow compensation: TR/TE = 2530/2.98 ms, voxel size = 1×1×1 mm³, slice thickness = 1 mm, matrix = 256×256, 192 slices, acquisition time ≈ 5–7 min. Both magnitude and phase images will be saved.

⑦Neuromelanin-sensitive MRI (NM-MRI): T1-weighted turbo spin-echo sequence, TR/TE = 600/13 ms, slice thickness = 2.5 mm, FOV = 220×220 mm², 16 axial slices oriented perpendicular to the brainstem, acquisition time = 8 min.

**2. Quality Control and Exclusion Criteria**

All raw images will be visually inspected by two experienced radiologists for artifacts. Images with severe artifacts will be excluded from analysis.

For rs-fMRI and DTI, framewise displacement (FD) will be calculated. Participants with >20% of volumes exceeding FD > 0.5 mm, or with maximum head motion > 3.0 mm translation or 3.0° rotation, will be excluded. For DTI, volumes with excessive motion will be removed using FSL’s eddy tool. T1-weighted images will be checked for adequate gray/white matter contrast. For fMRI, temporal signal-to-noise ratio (tSNR) will be computed; whole-brain mean tSNR < 50 will be considered for exclusion. For VBM, sample homogeneity will be assessed using covariance; outliers >2 standard deviations from the mean will be visually inspected and potentially excluded.

**3. Preprocessing and Analysis Pipelines**

**3.1 Structural MRI (VBM)**

T1-weighted images will be preprocessed using CAT12 (r1720) in SPM12 (r7771). Steps include bias correction, segmentation into gray matter (GM), white matter (WM), and cerebrospinal fluid (CSF), and spatial normalization to MNI152 space using DARTEL. Modulated GM volumes will be smoothed with an 8 mm FWHM Gaussian kernel. Voxel-wise general linear model will be used to compare GM volume between groups (PD vs. HC) and across time points, covarying for age, sex, and total intracranial volume. Results will be thresholded at voxel-level p < 0.001 (uncorrected) and cluster-level FWE-corrected p < 0.05.

**3.2 Resting-State fMRI**

Resting-state fMRI data will be preprocessed using DPABI v6.1. The preprocessing pipeline includes removal of the first 10 volumes, slice timing correction, realignment, and normalization to MNI space using deformation fields derived from T1 segmentation. Data will then be smoothed with a 6-mm FWHM Gaussian kernel, followed by detrending, nuisance regression (including Friston-24 motion parameters and WM/CSF signals), and bandpass filtering (0.01–0.08 Hz). For motion scrubbing, volumes with FD > 0.5 mm will be modeled as nuisance regressors (“censoring”/scrubbing regressors) rather than deleting the entire time series; participants with more than 20% censored volumes, or with maximum head motion >3.0 mm translation or 3.0° rotation, will be excluded.

Seed-based functional connectivity will be computed using seeds defined by the AAL atlas. MNI coordinates for each seed (center of mass) are: Putamen (left): [−25, 2, 2]; (right): [27, 2, 2], Caudate (left): [−13, 10, 10]; (right): [14, 10, 10], Globus pallidus (left): [−20, −2, 0]; (right): [22, −2, 0], Thalamus (left): [−12, −18, 8]; (right): [13, −18, 8]. Whole-brain voxel-wise inference will use cluster-level FWE correction (voxel-level p < 0.001; cluster-level p < 0.05). To control multiplicity due to testing multiple seeds, seed-level p-values will be further controlled using FDR (Benjamini–Hochberg) across seeds at q < 0.05. The bilateral putamen will be treated as the a priori primary seed, while the remaining seeds will be interpreted as secondary/exploratory under FDR control across seeds.

**3.3 Diffusion Tensor Imaging**

Diffusion tensor imaging data will be processed using DTIStudio v3.0.3. Eddy current and motion correction will be applied using affine registration to the b0 image, and the diffusion tensor will be estimated by linear least squares fitting to generate fractional anisotropy (FA), mean diffusivity (MD), radial diffusivity (RD), and axial diffusivity (AD) maps. Deterministic streamline tractography will be performed for the nigrostriatal pathway using a two-ROI approach, with the substantia nigra as the seed and the putamen/caudate as the target. Fiber tracking parameters include an FA threshold of 0.2, turning angle < 60°, and step length of 1 mm. For ROI-based analysis, regions of interest (substantia nigra, putamen, caudate) will be manually delineated on FA color maps by two independent raters, with inter-rater reliability assessed using intraclass correlation coefficient (ICC > 0.8 considered acceptable; discrepancies resolved by consensus). Mean FA, MD, RD, and AD values will be extracted from each ROI, and statistical comparisons will use paired t-tests or ANOVA with Bonferroni correction for the three ROIs.

**3.4 SWI and NM-MRI**

For susceptibility-weighted imaging (SWI), phase images will be processed using in-house MATLAB scripts incorporating high-pass filtering (32×32 kernel) to remove background field inhomogeneities, followed by phase mask multiplication to generate SWI images. Two independent raters will manually outline the substantia nigra (SN) area and measure the width of the SN pars compacta (SNc) on the axial slice showing the maximum SN area, using MRIcron. SNc width will be measured in the dorsoventral direction at the midpoint of the SN. For neuromelanin-sensitive MRI (NM-MRI), images will be viewed on the ADW4.6 workstation, and SNc width will be measured on the axial slice where the hyperintense SNc is best delineated from the hypointense SNr, with measurement performed perpendicular to the long axis of the SNc at its widest point. Both SWI and NM-MRI measurements will be performed independently by two raters, with inter-rater reliability assessed via ICC, and the average of the two measurements used for subsequent analysis. For statistical analysis, ROI values will be compared using ANCOVA (with age and sex as covariates) and Bonferroni correction applied for multiple ROIs (SN area, SNc width from SWI, and SNc width from NM-MRI).

**3.5 Healthy Control Data Analysis**

To establish PD-related brain alterations at baseline, healthy controls (HCs) data will be compared with PD patient data (all PD groups combined at baseline) using the following procedures. For whole-brain VBM and rs-fMRI seed-based connectivity analyses, two-sample t-tests will be performed in SPM12, with age and sex included as covariates. Results will be thresholded at voxel-level p < 0.001 (uncorrected) and reported at cluster-level FWE corrected p < 0.05. For ROI based analyses—including DTI metrics in predefined nigrostriatal ROIs (substantia nigra, putamen, caudate), susceptibility-weighted imaging (SWI)-derived substantia nigra area and SNc width, and NM-MRI-derived SNc width—two-sample t-tests or ANCOVA (covarying for age and sex) will be performed. Bonferroni correction will be applied based on the number of predefined ROIs within each modality.

**4. Software and Version Information**

SPM12 (r7771, https://www.fil.ion.ucl.ac.uk/spm/)

CAT12 (r1720, http://www.neuro.uni-jena.de/cat/)

DPABI v6.1 (http://rfmri.org/dpabi)

DTIStudio v3.0.3 (https://www.mristudio.org/)

MATLAB R2013b (MathWorks, Natick, MA)

NODDI toolbox (http://mig.cs.ucl.ac.uk/index.php?n=Tutorial.NODDImatlab)

MRIcron (https://www.nitrc.org/projects/mricron)

ADW4.6 workstation (GE Healthcare)

**5. Multiple Comparisons Correction Summary**

Whole-brain VBM and rs-fMRI: cluster-level FWE correction (voxel p < 0.001, cluster p < 0.05)

ROI-based DTI, SWI, NM-MRI: Bonferroni correction per ROI set (e.g., three ROIs → p < 0.05/3 ≈ 0.017)

All tests two-tailed, significance threshold α = 0.05 after correction.
